# Pharmaceutical Development and Safety Evaluation of a GMP-Grade Fucoidan for Molecular Diagnosis of Cardiovascular Diseases

**DOI:** 10.3390/md17120699

**Published:** 2019-12-12

**Authors:** Cédric Chauvierre, Rachida Aid-Launais, Joël Aerts, Frédéric Chaubet, Murielle Maire, Lucas Chollet, Lydia Rolland, Roberta Bonafé, Silvia Rossi, Simona Bussi, Claudia Cabella, Laszlo Dézsi, Tamas Fülöp, Janos Szebeni, Youssef Chahid, Kang H. Zheng, Erik S. G. Stroes, Dominique Le Guludec, François Rouzet, Didier Letourneur

**Affiliations:** 1 Université de Paris, UMRS1148, INSERM, F-75018 Paris, France; rachida.aid@inserm.fr (R.A.-L.); frederic.chaubet@univ-paris13.fr (F.C.); murielle.maire@univ-paris13.fr (M.M.); lucas.chollet35@gmail.com (L.C.); dominique.leguludec@gmail.com (D.L.G.); francois.rouzet@aphp.fr (F.R.); didier.letourneur@inserm.fr (D.L.); 2 X. Bichat Medical School, Université de Paris, UMS34 FRIM, F-75018 Paris, France; aerts@radiopharm.eu; 3 AP-HP, Department of Nuclear Medicine, X. Bichat Hospital, F-75018 Paris, France; 4 Algues & Mer, F-29242 Ouessant, France; lydia.rolland@algues-et-mer.com; 5 Bracco Research Center, Bracco Imaging Spa, 20811 Colleretto Giacosa, Italy; roberta.bonafe@bracco.com (R.B.); Silvia.Rossi@bracco.com (S.R.); Simona.Bussi@bracco.com (S.B.); Claudia.Cabella@Bracco.com (C.C.); 6 Nanomedicine Research and Education Center, Semmelweis University, 1085 Budapest, Hungary; dr.dezsi.laszlo@gmail.com (L.D.); fulopgyulatamas@gmail.com (T.F.); jszebeni2@gmail.com (J.S.); 7 Department of Vascular Medicine, Academic Medical Center, 1105 AZ Amsterdam, The Netherlands; y.chahid@amc.uva.nl (Y.C.); k.h.zheng@amc.uva.nl (K.H.Z.);

**Keywords:** GMP-grade fucoidan, regulatory toxicology, molecular diagnosis, scintigraphy

## Abstract

The adhesion molecule P-selectin is present on the cell surface of both activated endothelium and activated platelets. The present study describes the pharmaceutical development, safety evaluation, and preclinical efficacy of a micro-dosed radiotracer. The macromolecular nanoscale assembly consisted of a natural compound made of a sulfated fucose-rich polysaccharides (fucoidan) and a radionuclide (technetium-99m) for the detection of *P*-selectin expression in cardiovascular diseases. After extraction and fractionation from brown seaweeds, the good manufacturing practice (GMP) production of a low molecular weight (LMW) fucoidan of 7 kDa was achieved and full physicochemical characterization was performed. The regulatory toxicology study in rats of the GMP batch of LMW fucoidan revealed no adverse effects up to 400 μg/kg (×500 higher than the expected human dose) and pseudoallergy was not seen as well. In a myocardial ischemia-reperfusion model in rats, the GMP-grade LMW fucoidan labeled with technetium-99m detected *P*-selectin upregulation in vivo. The present study supports the potential of using ^99m^Tc-fucoidan as an imaging agent to detect activated endothelium in humans.

## 1. Introduction

Cardio- and neuro-vascular diseases are currently the leading cause of death world-wide, and their burden is likely to increase due to ageing of the population and the rising prevalence of metabolic disorders such as diabetes and obesity [1]. Despite the development of potent cardiovascular prevention regimens and early endovascular and surgical interventions, coronary artery disease (CAD), and stroke remains associated with high mortality and morbidity [2]. Since atherosclerosis is a silent and asymptomatic disease until its clinical sequelae, early diagnosis is mandatory to reduce cardiovascular risk and prevent irreversible damage. To that end, visualization of biological processes at cellular and molecular levels is needed. Molecular imaging is a promising tool based on a contrast agent designed to recognize a molecular target of interest and to bind it specifically [3]. Adhesion molecules, such as selectins, persist on the endothelial surface after ischemia has resolved. Therefore, they represent a tissue ‘imprint’ of the prior ischemic event, referred to as ‘ischemic memory’ [4]. P-selectin is an adhesion molecule expressed at the surface of endothelial cells and platelets upon activation. It mediates leukocyte rolling on activated endothelium [5] and leukocyte trapping on platelet aggregates [6], through the interaction with its counter receptor *P*-selectin glycoprotein ligand 1 (PSGL-1). The interaction between *P*-selectin and PSGL-1 involves the tetrasaccharide SLeX [7,8,9]. Many studies have shown that mimics of SLeX, oligosaccharides, and sulfated polysaccharides such as heparin, heparan sulfate, dextran sulfate, and fucoidan or some of their derivatives, were able to interact with *P*-selectin [10,11,12]. Among them, fucoidans are a family of polysaccharides extracted mainly from brown algae and containing a substantial percentage of l-fucose and ester sulfate. We showed that low molecular weight fucoidan prevented *P*-selectin binding to SLeX with an IC_50_ of 20 nM and exhibited a high affinity for immobilized *P*-selectin with a *K*_D_ of 1.2 nM. Moreover, the binding of fucoidan to human platelets increased with the level of platelet activation, and the binding of anti *P*-selectin antibody to activated platelets was inhibited by fucoidan, demonstrating the specificity of the interaction between fucoidan and *P*-selectin [13].

Since nuclear imaging with single-photon emission computed tomography (SPECT) allows for in vivo molecular imaging with high sensitivity, we propose ^99m^technetium-fucoidan as a new molecular contrast agent with high specificity for *P*-selectin. In the current study, we completed full pharmaceutical development of ^99m^Tc-fucoidan to assess its molecular diagnostic efficacy and its safety profile of cardiovascular diseases before entering a future phase I clinical trial.

## 2. Results and Discussion

### 2.1. Composition of Low Molecular Weight Fucoidan as Active Pharmacological Ingredient (API)

The present purified extract is under investigation for application in human health for molecular diagnosis. With reference to the Ph. Eur. 07/2015:0765 on herbal drug extract, purified fucoidans is classified as ‘other extract’ as it is neither adjusted to a defined content of one or more constituent with known therapeutic activity (i.e., standardized) nor adjusted to one or more active markers (i.e., ‘quantified’). The extract is defined by the minimum level of fucoidan (constituted of fucose and sulfate as markers) of 40% (w/w). Here, the fucoidan content in the API is 53.4% as is and 58% dried (Table 1). Fucoidan is obtained from *Ascophyllum nodosum* by extraction with water from the whole algae. It is a sulfated fucose-rich polysaccharide depolymerized to obtain low molecular weight polymers, here 7.1 kDa. The API composition in mass is based on a very low proportion of amino-acids (<0.75%), sulfates (21.7%), fucose (31.7%), galactose (2.8%), xylose (10.5%), mannose (3.7%), uronic acids (18%), calcium (1.8%), sodium (3.9%), potassium (0.8%), and loss on drying (8%). The mass balance is close to 100% so the API composition in main constituents is elucidated. Moreover, the API is stable since its content of free sulfate and free fucose is very low (0.1 and < 0.1% respectively). Considering inorganic impurities, heavy metals may come from the alga itself and from the manufacture process, especially arsenic, mercury, lead, and cadmium. Analysis of the API have shown that most of heavy metals are widely below the stated specification limits (Table 1). Formaldehyde is used in first step of the process in order to block phlorotannins in algae. Algae are rinsed off in water in order to eliminate formaldehyde but residual traces of the solvent can be present in the API. The formaldehyde content of the API is lower (170 ppm) than the acceptance limit (200 ppm). Finally, the potential bacterial contamination sources can be algae (harvest and storage), raw materials, equipment, and personnel. Quality controls and GMP reduce the risk of contamination. Therefore, the API is free from bacterial endotoxins and specified micro-organism such as *Pseudomonas aeruginosa, Staphylococcus aureus, Bacillus subtilis*, and *Escherichia coli.*

**Figure 1:**
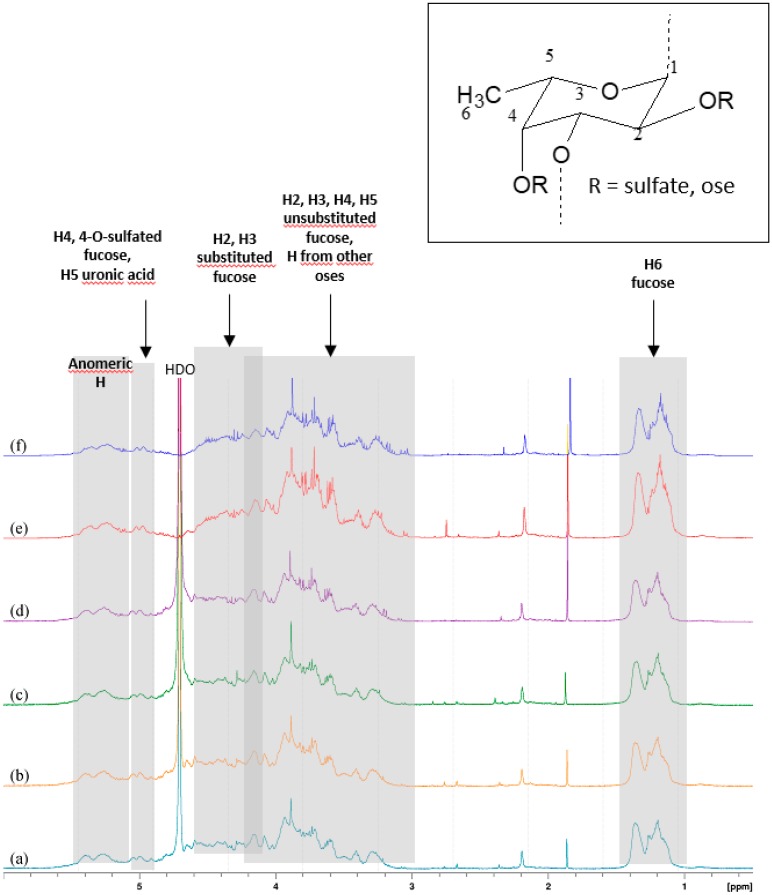
1D-^1^H-NMR spectra of 6 API batches.

The ^1^H-NMR spectra of the active pharmacological ingredient (API) confirmed the predominant presence of alpha-1→3 bonds between the glycosidic units. Since it remains a mixture spectrum appeared too complex to deduce precise and relevant information on the structure or sequence of the residues. However, 1D spectra made it possible to highlight certain characteristics (Figure 1). Resonance signals of the anomeric protons of α-l-fucose and some side oses would appear between 5.08 ppm and 5.50 ppm. Between 4.90 ppm and 5.10 ppm we observe two groups of signals, likely from H-4 fucose units sulfated at *O*-4 and H-5 from uronic acids. From 4.10 ppm to 4.66 ppm, the H-2 and H-3 protons of the substituted fucose, and between 3.00 ppm and 4.20 ppm the H-2, H-3, H-4, and H-5 non-sulfated fucose units form a complex set of signals. In this region, signals from the other oses in smaller amounts are masked by those of fucose. From 1.20 ppm to 1.36 ppm appear the H-6 protons of the methyl groups of fucose [14,15,16,17]. The identification of the glycosidic structures responsible for the interaction with *P*-selectin and of those implicated in the complexation of technetium ions is underway and will subsequently be the subject of a dedicated publication.

### 2.2. Composition of GMP-Grade LMW Fucoidan (GMP Batch)

The objective of the pharmaceutical development was to develop a sterile drug product physically and chemically stable taking into account the properties of the drug substance (fucoidan) and the intended route of administration (intravenous). A simple stable freeze-dried formulation including sodium chloride as matrix has been selected. The freeze-dried fucoidan is intended to be reconstituted with other ingredients (ascorbic acid, stannous chloride) and radiolabeled before administration. The GMP batch is a lyophilized powder containing 40 µg of fucoidan as drug substance and 8 mg of NaCl as dispersing agent. The GMP batch is filled in 6*R* (9 mL) clear type I glass vial closed with a FluroTec^®^ stopper, sealed with an aluminum cap and blue flip-off capsule. Manufacture and quality control of GMP batch for the clinical study were performed in compliance with good manufacturing practice at dully authorized facilities. Further steps of reconstitution and radiolabeling are carried out at the hospital. The GMP batch is a white powder with 2.3% (m/m) of water content. The reconstituted solution in water is clear and colorless with 18 sub-visible particles higher than 10 µm and no one higher than 25 µm per container. The GMP batch exhibited a fucoidan content of 40.3 µg (i.e., 100.8% of nominal content) and a very low content in free fucose (<0.1%). No interference between the fucoidan and the other ingredient (NaCl) is expected. Finally, the GMP batch complied with the required microbiological quality (no microbial growth and absence of bacterial endotoxins) (Table 2).

### 2.3. Stability of GMP Batch

Stability study has been performed on GMP batch. Since no change in tested quality criteria was observed after 12 months of storage under long-term (25 °C–60% RH) (Table 3) and after 6 months under accelerated conditions (40 °C–75% RH) (Table 4), according to ICH standards a provisional shelf-life of 24 months is currently proposed. This shelf-life will be extended according to the further results of the ongoing long-term stability study.

### 2.4. Composition of the Radiolabeled GMP-Grade LMW Fucoidan (Investigational Medicinal Product)

The aim of the pharmaceutical development was to obtain a simple reconstitution procedure from usual excipients mostly used for radiolabeling with technetium-99m (^99m^Tc). For the purpose of the labeling, an interaction between a ^99m^Tc form and the chain of fucoidan must occur. The ions ^99m^TcO_4_^−^ must be reduced in order to free positions of coordination on the metal and promote the ionic interactions with the negative chain of the polysaccharide (sulfate groups). After reduction from the +VII state in the pertechnetate ion, different forms are known: Tc_(V)_O^3+^, Tc_(V)_O^2+^, Tc_(I)_^+^. Stannous ions Sn_2_^+^ were used to obtain the reduction. Optimal conditions were selected: 40 µg of lyophilized fucoidan as API, 1000–1300 MBq of ^99m^Tc sodium pertechnetate, 12 µg stannous chloride dehydrate (corresponding to 10 µg of anhydrous stannous chloride) as reducing agent, 10 µg ascorbic acid as antioxidant, 21.5 mg of NaCl for isotonicity agent (coming from fucoidan lyophilized powder (8 mg), saline solution of ^99m^Tc (4.5 mg) and saline solution used during the preparation process), and 2 mL of water for injection.

Radioactivity levels on three independent experiments were of 758, 546, 592 MBq, with purity of 96, 95, and 96% respectively with 1–3% of non-reduced technetium or radioactive colloids (Table 5). During the stability test, the radiochemical purity remained >95% up to one hour after filtration, and decreased thereafter in relation to the formation of colloids.

Based on the results, the reconstituted radiolabeled solution for injection can be used up to one hour after filtration. It should be kept at room temperature in lead protection (no exposition to light).

As reported in Table 5, the recovered activity after filtration is subjected to some variability due to multiple causes. First, the same volume of pertechnetate eluate is used for every test (500 microliters). The volumic activity at generator elution changes from one test to another. Secondly, the yield of radiolabeling and the amount of activity trapped on filters after double filtration is not the same in every test. All the presented results are in accordance with radioactivity specifications.

After reduction by tin chloride, the proposed mechanism of labeling is an interaction between a reduced form of technetium-99m with the negative charge of fucoidan (sulfate) and/or with the hydroxyl moieties. The exact level of reduction and the structure of interaction are unknown.

In this study, the labeling of the natural polysaccharide fucoidan was done without modifying the chemical structure of the polymer in order to obtain a radiolabeled macromolecular assembly at nanoscale for molecular diagnosis by scintigraphy of *P*-selectin expression in cardiovascular diseases.

### 2.5. Acute Oral Toxicology of GMP Batch

No mortality occurred following dosing in rats (*n* = 5). No adverse signals were observed during the 14 days post-dosing. Changes in body weight observed during the period of the study were within the range expected for this strain and age of animals. No abnormalities were found on necropsy of animals on termination of the study. The results of this study indicate that the test substance LMW fucoidan has no observable toxic effect following oral administration of a single dose at a level of 2000 mg/kg.

### 2.6. Mutagenicity of GMP Batch

No evidence of toxicity was observed at any dose level tested (50–5000 micrograms/plate), in the absence or presence of S9 metabolic activation with tested strains. For the test substance to be considered mutagenic, two-fold (or more) increases in mean revertant numbers must be observed at two consecutive dose-levels or the highest practicable dose-level only. In addition, there must be evidence of a dose–response relationship showing increasing numbers of mutant colonies with increasing dose-levels. The effect must be reproduced in an independent experiment. The test substance did not induce increases in the number of revertant colonies which were two-fold greater than the control values at any dose-level, in the absence or presence of S9 metabolism. On the basis of the stated criteria, it must be concluded that the LMW fucoidan is not mutagenic to *Salmonella typhimurium* under the reported experimental conditions.

### 2.7. Biodegradability of GMP Batch

Oxygen consumption, and thus the derived BOD, showed low degradation of the substance. The toxicity control containing both the test substance and the reference substance, showed less than 25% degradation (based on total ThOD) within 14 days indicating that the test substance was inhibitory to the test system. 

### 2.8. Extended Single Dose Toxicity Study of GMP Batch after Intravenous Administration to Rats

This study was performed to determine the toxicological profile of fucoidan extract formulation after the intravenous administration to rats at three different doses: 40, 250, and 400 μg/kg in comparison to a control group administered with physiological saline. 

No animals died during the study and no clinical signs or treatment related changes in body weight or body weight gain were observed in all animals treated with fucoidan extract formulation up to 400 µg/kg. There were no test item-related changes in body weight, hematology, blood chemistry, coagulation, and urinalysis parameters of animals—either males or females—sacrificed 24 h or 14 days after exposure to the fucoidan extract formulation.

No treatment related changes in absolute and relative organ weights were observed and no fucoidan extract-related microscopic findings were observed at histopathology.

Based on these results, it can be concluded that the NOAEL for fucoidan extract formulation after a single intravenous administration to rats is 400 μg/kg.

### 2.9. Effect of GMP Batch on Complement Activation Related Pseudoallergy (CARPA) in a Hypersensitivity Model in Pigs

**Figure 2:**
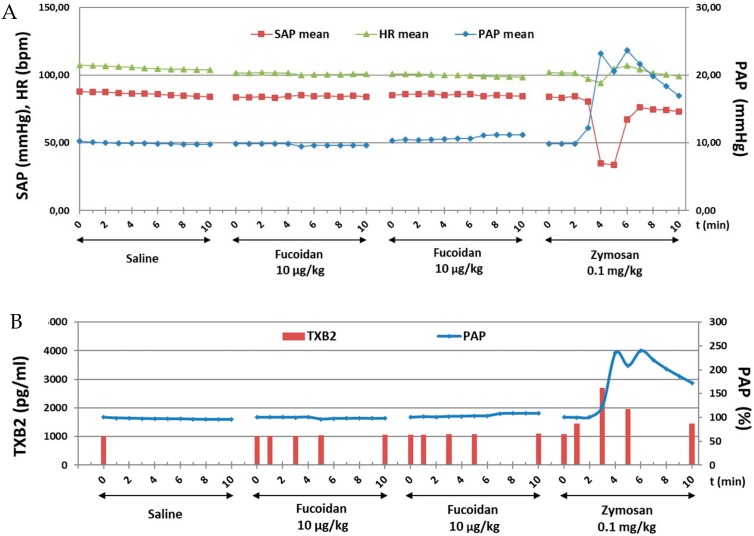
Time course of cardiovascular (PAP, SAP and HR) changes (**A**) and changes in PAP and plasma TXB levels (**B**) following fucoidan and zymosan *i.v.* injections in pigs.

This study investigated the immune reactive properties of GMP-grade LMW fucoidan in a highly sensitive model of CARPA in pigs. Cardiopulmonary effects of vehicle (saline control) and repeated bolus administrations of 10 µg/kg i.v. of fucoidan are shown. None of the boluses, led to any change in PAP, SAP, and HR. At the end of the experiment 0.1 mg/kg i.v. zymosan induced CARPA denoted by a 2.5-fold increase in PAP, and a strong transient reduction (by more than 50%) in SAP (Figure 2A). Plasma TXB2 assay indicated that fucoidan induced no thromboxane production while zymosan caused 2.7-fold increase in TXB2 in parallel with PAP elevation (Figure 2B). Fucoidan in 1 μg/kg (data not shown) and 100 μg/kg (Figure S1) did not show any CARPA.

### 2.10. In Vivo Myocardial Ischemia-Reperfusion Results

**Figure 3:**
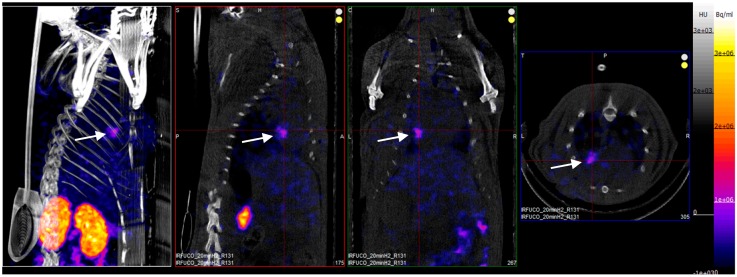
SPECT/CT scans of an ischemia-reperfusion rat model acquired 2 h after intravenous injection of GMP grade LMW fucoidan labeled with ^99m^Tc (Investigational Medicinal Product) (white arrows). Representative whole body SPECT/CT imaging: from left to right: 3D view, sagittal, coronal, and axial planes.

Four hours after reperfusion, a clear focal uptake of GMP grade LMW fucoidan labeled with ^99m^Tc (Investigational Medicinal Product) had been detected in all animals confirming the presence of exposed *P*-selectin [18], either by endothelium cells or by activated platelets in the area of myocardial ischemia (Figure 3).

## 3. Materials and Methods

### 3.1. Low Molecular Weight Fucoidan (Active Pharmacological Ingredient)

**Figure 4:**
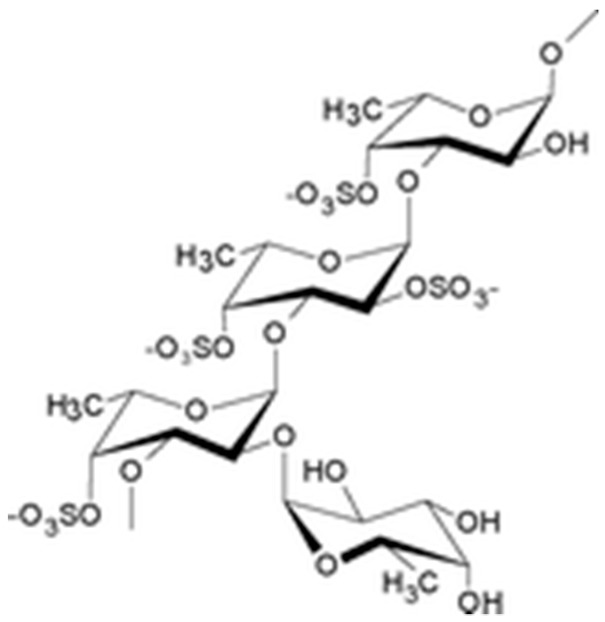
Schematic structure of fucoidan.

The drug substance is a lyophilized powder containing low molecular weight (LMW) fucoidan, a sulfated polysaccharide (Figure 4) extracted with water from the fresh algae *Ascophyllum nodosum*. Crude extract of LMW fucoidans is marketed by Algues & Mer (France) under Ascophyscient^®^ trade name (CAS#84775-78-0). Algues & Mer harvests the seaweeds on the foreshore, between the high water of the neap tide and the mid-tide, mostly on the sheltered area. GPS cartography is used to collect the alga parcel after parcel, in a sustainable way. Freshly harvested parcels are not exploited for at least 3 years. Harvest is run by hand cutting, 30 cm above the seaweed holdfast. Fucoidan from high molecular weight extracts was concentrated on 100 kDa membranes and depolymerized to obtain low molecular weight polymers (between 5–10 kDa). These polymers were filtrated and purified with a 3 kDa membrane. The methods of the fucoidan depolymerization and purification are industrial processes from Algues & Mer. For proprietary reasons, these methods cannot be disclosed in details here. Anyway, fucoidan is reproducibly extracted from the alga *Ascophyllum nodosum* by the company Algues & Mer. Its degradation is carried out by an oxydative-reductive process (ORD) to effectively reduce the molar mass of raw macromolecular species. This degradation is carried out non-selectively with respect to the glycosidic bonds (which is not the case for acid hydrolysis) in a yield greater than 50% by weight. Its purification is carried out by ultrafiltration. The low molecular weight fucoidan obtained is not a single molecule but a mixture of macromolecular species, of reproducible composition as demonstrated by the analysis of many batches [19]. A steric exclusion chromatogram is provided in Figure S2. So is the Active Pharmacological Ingredient which average composition is reported in the manuscript after purification following the recommendations of the European Pharmacopoeia.

The yield of the LMW fucoidan from the alga *Ascophyllum nodosum* after depolymerization and purification steps was 5% (*w*/*w*).

### 3.2. Controls of LMW Fucoidan

#### 3.2.1. Molecular Weight Determination

The absolute average molecular weight was determined at room temperature by coupling online a high-performance size exclusion chromatograph (HPSEC), a multi-angle laser light scattering detector (MALLS), and a differential refractive index (dRI) detector. 0.1 M LiNO_3_, used as carrier, was filtered through a 0.1 μm, degassed and eluted at a 0.5 mL/min flow rate (LC10Ai Shimadzu, Kyoto, Japan). The SEC line consisted of an OHpak SB-G guard column for protection and two OHpak SB-802.5 and -803 HQ columns (Showa Denko Europe, Munich, Germany) in series. The MALLS photometer, a DAWN HELEOS II from Wyatt Technology Inc. (Santa Barbara, CA, USA) was provided with a fused silica cell and a Ga-As laser (*λ* = 665.8 nm). Molar mass was obtained with the Zimm order 1 method using angles between 53.1° and 140°. The concentration of each eluted fraction was determined with dRI (RID10A Shimadzu, Kyoto, Japan) according to the measured values of *d_n_*/*d_c_* (0.144 mL/g).

#### 3.2.2. Determination of Total and Free Sulfates 

The total sulfates were quantified by ion exchange chromatography coupled with conductivity detection against a standard solution (from sodium sulfate) after acid hydrolysis. The settings were: column (Anion Exchange IonPac 250 × 4.0 mm, 4.0 µm particle size); flow rate (1 mL/min); injection volume (10 µL); detection (conductivity with cation suppressor); column and sample temperatures (25 °C); mobile phase (12 mM NaOH); run time (10 min); and gradient (isocratic). Determination of free sulfates is carried out before hydrolysis step: the free sulfate is an analytical marker of the stability of the active substance.

#### 3.2.3. Assay of Total Fucose, Other Monosaccharides, and Free Monosaccharides 

The assay of fucoidan is based on the total fucose determination by high performance anion exchange chromatography coupled with pulsed amperometry detection after acid hydrolysis. The settings were: column (Anion Exchange CarboPac 250 × 4.0 mm, 4.0 µm particle size); flow rate (1 mL/min); injection volume (10 µL); detection (pulsed amperometric detection); working electrode (gold); reference electrode (Ag/Cl); waveform (carbohydrate, quad potential); column and sample temperatures (25 °C); mobile phase (1 mM KOH); run time (15 min); and gradient (isocratic). Other monosaccharides (galactose, xylose, and mannose) are quantified as a fingerprint of the composition of the active substance in sugars. Determination of free sugars is carried out before hydrolysis step: the free fucose is an analytical marker of the stability of the active substance.

Loss on drying: according to Ph. Eur. 2.2.32 (Method D) with sample weigh of 1 g at temperature of 105 °C.

Total Ash: according to Ph. Eur.2.4.16.

Heavy Metals: heavy metals are analyzed by ICP-MS according to Ph. Eur. 2.4.27/2.2.58.

Microbiological quality: microbiological quality is controlled according to Ph. Eur. 2.6.12 for total aerobic microbial count (TAMC) and total combined yeasts and molds count (TYMC) as well as Ph. Eur 2.6.13. for specified micro-organisms.

Nuclear magnetic resonance: all experiments were conducted on a Bruker AVANCE III spectrometer (BioSpin Bruker, Wissembourg, France) operating at a proton frequency of 500 MHz with a 5 mm gradient indirect detection probe, at a probe temperature of 300 K. The samples were exchanged twice with 99.8% D_2_O with intermediate freeze drying and dissolved in 0.6 mL of 99.96% D_2_O. 1-d proton spectra were acquired with 16 scans and 32 K data points with a spectral width of 5000 Hz. Typical ^1^H 9.5 μs-pulse length and relaxation delay of 1 s were used. Water signal was suppressed by a presaturation sequence at the water signal frequency.

### 3.3. GMP Production of LMW Fucoidan (GMP Batch)

The manufacturing process consisted of the preparation of the fucoidan solution which was sterile filtered and aseptically filled into vials and finally freeze-dried. The manufacturing process consisted of seven consecutive steps in a class C room including the three last ones in isolator class A, and in-process controls and validation steps: (1) Dissolution of fucoidan with NaCl; (2) mixing; (3) 0.22 µm clarifying filtration; (4) 0.22 µm sterilizing filtration; (5) aseptic filling and pre-stoppering; (6) freeze-drying; (7) final stoppering, capping-, and crimping. The drug product is filled into 9 mL clear type I glass vials closed with the following primary packaging: a clear type I-glass vial 6*R*, a rubber stopper type I (FluroTec^®^, 13 mm diameter formulation 4023/50 grey B2-40 coating Westar^®^) (WEST), a flip off aluminum seal and a blue cap (all from Adelphi).

### 3.4. Controls of GMP-Grade LMW Fucoidan (GMP Batch)

Appearance: visual control.

Water content: according to E.P. 2.5.32 (coulometry).

Appearance of the solution: content of one vial was solubilized in 2 mL of water for injection. Visual control on the solution was performed.

Sub-visible particles: according to Ph. Eur. 2.2.19. Content of each vial was solubilized in 2 mL of water free of particles. Analysis was made on a pool of numerous vials.

Assay of fucoidan and free fucose: the fucose was assayed by high performance anion exchange chromatography coupled with pulsed amperometry detection (HPAEC-amperometry) after acid hydrolysis, as a marker of the fucoidan content in the drug product. The free fucose was determined using the same method, but before acid hydrolysis. The settings were the same than those for the assay of total fucose. The free fucose was a marker of the degradation of the fucoidan in the drug product.

Calibration standard preparation for fucose determination (free and total): a stock solution of fucose reagent >99% (Sigma-Aldrich, Saint Louis, MO, USA, ref. F2252) at 1 mg/mL was prepared as follow: into a 10 mL tube, 10 mg of fucose powder were accurately weighed (recording the exact weight), 10 mL of MilliQ water were added (adjusting the volume according to exact weight), well mixed, labeled “Fucose 1 mg/mL”, dated and paraphed. Dilutions with MilliQ water were prepared, ranging from 10 µg/mL to 0.1 µg/mL.

GMP batch preparation for assay of active substance: GMP batches were received in a freeze-dried powder form into a sealed vial. The following steps to prepare the drug product before analysis were applied: the seal was removed from the vial, 1 mL of MilliQ water was carefully added into the vial, well mixed, and visually checked to assure that all powder was dissolved. 400 µL of the vial content were transferred into a screw cap 2 mL microtube, added with 100 µL of hydrolysis solution and incubated 4 hours in a dry bath at 99 °C. After incubation, once the microtube has cooled down to room temperature, 400 µL of the hydrolysate was dispensed into a 1.8 mL glass vial and added with 400 µL of MilliQ water; the vial was labeled and placed it to the autosampler.

For free fucose determination, solution of reconstituted vial was directly injected into the HPAEC system.

Calculation of active substance (fucoidans) in GMP batch: the active substance batch used for the manufacture of the GMP batch had been characterized as follows (batch FUCO16388: 31.68% (w/w) fucose content): the quantity of fucose expressed as µg/vial of drug product was obtained from the concentration in µg/mL (given by the regression line) corrected for the dilution factor of the sample solution. The quantity of fucose analyzed in GMP batch was divided by 31.68% to obtain the quantity of active substance.

Sterility: according to Ph. Eur. 2.6.1.

Bacterial Endotoxins: according to Ph. Eur. 2.6.14 (Method D).

Stability: the study was conducted with following conditions on the GMP batch:

For long term storage conditions, the temperature was 25 ± 2 °C, the relative humidity was 60% RH ± 5 °C and the stability points were T1, T3, T6 and T12 months.

For accelerated storage conditions, the temperature was 40 ± 2 °C, the relative humidity: 75% RH ± 5 °C and the stability points were T1, T3, T6 months.

The test criteria were appearance; water content; appearance of the reconstituted solution in water; sub-visible particles; fucoidan content (assay of active substance); free fucose content (degradation products); sterility; and endotoxins.

### 3.5. Radiolabeling of GMP Batch (Investigational Medicinal Product)

The Investigational Medicinal product batch was prepared extemporaneously with a solution of stannous chloride and ascorbic acid and radiolabeled with a saline solution of technetium-99m (^99m^Tc). Tc-99m is obtained from a Mo-99/Tc-99m generator as a sterile solution of sodium [Tc-99m] pertechnetate in saline (Na^99m^TcO_4_ in NaCl 0.9%). The main steps in the extemporaneously preparation of labeled fucoidan were: (1) preparation of a solution of tin(II) chloride and ascorbic acid in water for injection (60 mg/L and 50 mg/L respectively); (2) elution of the Mo-99/Tc-99m generator, MA n°564.454-1 (GE healthcare) and withdrawing of ^99m^TcO_4_^−^ activity (around 1200 MBq in 500 µL); (3) addition of ^99m^TcO_4_^−^ solution in the fucoidan 40 µg vial. Dissolution of the dispersion; (4) addition of 200 µL of the tin (II) chloride and ascorbic acid (the time of incubation has been set to 10–15 min at room temperature); (5) withdrawing and dilution at 1 mL with water for injection of the labeling mixture; (6) double 0.2 µm filtration in grade A environment (Figure S3) The composition of the radiolabeled GMP-grade LMW fucoidan (investigational medicinal product) is presented in Table S1.

Radioactivity: for dose calibrator measurement, current procedures of the Nuclear Medicine Laboratory at Bichat Hospital were used. In summary, the sample to assay was introduced in the chamber of the dose calibrator using a specific rack with specified position for each type of geometry. A specific programed button for each type of couple isotope/geometry was selected and the value in MBq was displayed by the device and recorded by the operator.

Radiochemical purity (RCP): the radiochemical purity of ^99m^Tc-fucoidan was controlled before administration to patient by thin layer chromatography (TLC) by the following procedure.

Evaluation of the radiochemical purity (RCP) of labeled fucoidan has focused on two classical impurities for labeled technetium derivatives: non-reduced technetium and radioactive colloids using methyl-ethyl-ketone, MEK (2-butanone, Sigma-Aldrich, 443468) and ACD-A (anticoagulant-citrate-dextrose solution USP formula A, pH = 5, Macopharma) respectively.

The determination of percentage of non-reduced technetium was performed using plates (ITLC-SG Agilent Technologies SGI0001, 10 cm), MEK as eluent, 2 µL for the spots and a distance of elution of 8 cm and detection by TLC scanner MiniGita Raytest. 

The % of non-reduced technetium (A) = % of integrated activity for peak at *R*_f_ = 1.

(*R*_f_ (TcO_4_^−^) = 1; *R*_f_ (colloids) = 0; *R*_f_ (Tc-fucoidan) = 0).

The determination of percentage of radioactive colloids was performed using plates (ITLC-SG Agilent Technologies SGI0001, 10 cm), ACD-A as eluent, 2 µL for the spots and a distance of elution of 8 cm and detection by TLC scanner MiniGita Raytest.

The % of radioactive colloids (B) = % of integrated activity for peak at *R*_f_ = 0.

(*R*_f_ (TcO_4_^−^) = 1; *R*_f_ (colloids) = 0; *R*_f_ (Tc-fucoidan) = 1).

Radiochemical purity: RCP = 100 − A − B %

The *R*_fs_ of fucoidan in the two TLC systems were first determined using a non-radiolabeled solution. An example of ITLC-SG plates spotted with a solution of LMW fucoidan-FITC in water is reported in Figure S4.

Stability: the stability of the radiolabeled solution (^99m^Tc-fucoidan) has been studied up to 120 min after filtration on two batches by studying the radiochemical purity over time in the two different TLC systems (MEK, ACD-A).

### 3.6. Acute Oral Toxicology of GMP Batch

The procedures described in OECD guideline n°401 adopted 24 February 1987 was used.

Limit test ≤ 2000 mg/kg. Species/strain: rat Sprague Dawley. Vehicle: water.

### 3.7. Mutagenicity of GMP Batch

The experiments were performed using the procedures developed by AMES et al. [20] and revised by Maron and Ames [21]. In addition, the study was designed to comply with the experimental methods indicated in EEC Council Directive 92/69, part B; OECD Guideline for the Testing of Chemicals no. 471; TSCA Test Guideline, 40 CFR 798.5265.

The bacteria/strains were *Salmonella tiphymurium* TA1535 TA1537 TA98 TA100 TA102.

Solvent was sterile distilled water.

The concentration range in the main test: with or without metabolic activation: 313–5000 µg/plate. The concentration of the test substance observed to be toxic to bacteria: (a) In a preliminary test with or without metabolic activation: >5000 µg/plate. (b) In the main test with or without metabolic activation: >5000 µg/plate.

Metabolic activation was performed with two batches of S9 tissue homogenates. They were prepared from the livers of 5 young male Sprague Dawley rats which had received prior treatment with Phenobarbital and betanaphthoflavone to induce high levels of xenobiotic metabolizing enzymes.

### 3.8. Biodegradability of LMW GMP Batch

The procedures used complied with the test for ready biodegradability described in OECD Guideline no. 301F adopted on 17 July 1992. Methods were in agreement with those of Commission Directive 92/69/EEC. The reference substance was sodium acetate.

### 3.9. Extended Single Dose Toxicity Study of GMP Batch after Intravenous Administration to Rats

The aim of this study was to determine the possible toxic effects of fucoidan extract formulation, after a single intravenous administration in rats, in order to identify the dose corresponding to the NOAEL (No Observed Adverse Effect Level). The ICH M3 (R2) guideline was applied (guidance on nonclinical safety studies for the conduct of human clinical trials and marketing authorization of pharmaceuticals [CPMP/ICH/286/95]).

The study was performed at the (1) Test Facility of Bracco Imaging SpA, via Ribes 5, 10,010 Colleretto Giacosa (TO); (2) Test Site: Charles River Laboratories, Tranent, Edinburgh EH33 2NE, United Kingdom (Histological processing and microscopic examinations), in compliance with National and International Good Laboratory Practice (GLP) regulations.

The animal study was performed in respect of the applicable regulation for animal experimentation and with approval of Italian Ministry of Health (research project no. 16/2015-PR).

Experimental design: seven-week-old Crl:CD^®^ (SD) BR rats of both sexes, acclimated for a week before the experiment, were treated intravenously, via tail vein, with three different doses of fucoidan extract formulation (40, 250, or 400 μg/kg). A control group was injected with physiological saline.

On the basis of body surface area (BSA), according to the extrapolation factor set for rats in FDA human equivalent dose (HED) guidance, these doses correspond to about 8, 50, and 80-fold the expected clinical dose of 40 μg/patient, respectively.

Animals were sacrificed at two time points: one early after administration (on day 1, interim sacrifice; 10 animals/sex/group), the other at the end of observation period (day 14, final sacrifice; 5 animals/sex/group).

All animals were observed for mortality and clinical signs immediately after the treatment and at different times during the day. Interim sacrifice animals were also observed on Day 1, before sacrifice while final sacrifice animals were inspected once a day during the 14-day off-dose period, until necropsies.

The day before scheduled sacrifice, all animals were fasted overnight (receiving a gavage of 15 mL/kg of tap water) in metabolic cages for about 16 h in order to obtain urine samples.

Subsequently, animals were anesthetized with SevoFlo at an induction rate of 3–4% followed by intramuscular injection of Zoletil^®^ 100 (20 mg/kg; Virbac) and Rompun^®^ (5 mg/kg; Bayer, Germany) and blood was collected after cannulation of the abdominal aorta for hematology, clinical chemistry and coagulation analyses.

Organs and tissues were processed from all animals and evaluated microscopically after staining with hematoxylin and eosin at the Test Site Charles River Laboratories.

Test item formulation: the 20 μg/mL solution was prepared by adding 2 mL of water for injection to each vial containing 40 μg of fucoidan extract, batch PF.797.IS, before administration. The stability of the reconstituted formulation was 1 week at room temperature. The concentration of the solution was checked and confirmed by Algaia, the actual concentration being about 10% higher than the nominal one.

Data analysis: qualitative analysis of mortality, clinical signs, urinalysis, and gross lesions was performed for all animals. Statistical analysis was performed on all the other recorded parameters, divided per sex, to compare all groups versus control group, and all groups each other’s, at two different sacrifice time points, using SPSS 24.0.

### 3.10. Effect of LMW GMP Batch on Complement Activation Related PseudoAllergy (CARPA) in a Hypersensitivity Model in Pigs

Domestic male Yorkshire pigs (20–25 kg) were sedated with ketamine/xylazine (10 and 2 mg/kg respectively) and anesthetized by isoflurane (2–3% in O_2_). Animals were breathing spontaneously. Pulmonary arterial pressure (PAP) was measured using a Swan-Ganz catheter introduced into the pulmonary artery via the right external jugular vein, while systemic arterial pressure (SAP) and heart rate (HR) were measured in the femoral artery. The left femoral vein was cannulated for blood sampling. All test agents were injected in bolus (<10 sec) via the left external jugular vein (*n* = 2–3/group). Hemodynamic changes were continuously monitored using an AD Instruments (ADI) PowerLab System. Mean PAP, SAP, and HR data were evaluated by the ADI LabChart software. 

Plasma Thromboxane B2 (TXB2—the stable metabolite of plasma TXA2) levels were measured with an ELISA kit (Cayman Chemicals).

Fucoidan was given in several experiments in a dose ranging from 1 to 100 μg/kg, single or repeated. Here one typical experiment’s data is shown only. To induce CARPA, zymosan (a glucan from yeast cell walls) was used for direct complement activation.

### 3.11. In Vivo Molecular Diagnosis in Myocardial Ischemia-Reperfusion Model on Rats

The animal study was performed in respect of the applicable regulation for animal experimentation and with approval of the animal care and use committee of the Claude Bernard Institute (APAFIS no. 2018060414026033, Paris, France).

Male Wistar rats (Janvier, France) were used in this study. Rats were anesthetized with isoflurane gas. Rat tracheas were cannulated to provide artificial respiration with a ventilator. A thoracotomy was performed between the third and the fourth intercostal space and the pericardium was incised. A 6.0 thread needle (Prolene™, Ethicon, Somerville, NJ, USA was passed around the left anterior descending coronary artery near its origin. Both ends of the thread were passed through a 1 cm long catheter (outer diameter, 2.0 mm), which was used to ligate the coronary artery. After 20 min of ischemia, reperfusion was conducted by cutting the thread and the chest cavity was then closed with stitches (3.0, Ethicon, Somerville, NJ, USA). 

70 MBq radiolabeled fucoidan was injected (penis vein) after 2 h of reperfusion. The SPECT/CT images were acquired 2 h after injection, using a nanoSPECT/CT apparatus (Mediso 30 medical imaging systems, Hungary) with a four-headed multiplexing multipinhole camera. Each head was equipped with a tungsten collimator (rat whole body—high sensitivity).

Statistical analysis: All results are presented as mean ± standard error of the mean (*n* ≥ 3).

## 4. Conclusions

We report here the pharmaceutical development and preclinical evaluation of a micro-dosed radiotracer based on the macromolecular assembly at nanoscale of a natural compound extracted from brown algae (*Ascophyllum nodosum*) consisting of a sulfated fucose-rich polysaccharides (fucoidan) and a radionuclide (technetium-99m) for the molecular diagnosis of cardiovascular diseases. After optimizing the extraction, depolymerization and purification processes, we obtained the monographs of the raw material (API) and the GMP batch with a full characterization of the main constituents since the mass balance is close to 100%. As the investigational medicinal product is a micro-dosed radiotracer for scintigraphy, our pharmaceutical development used a simple reconstitution procedure based on the mixture of the GMP batch with a solution of stannous chloride and ascorbic acid and radiolabeled with a saline solution of technetium-99m. The radiochemical purity remained higher than 95% up to one hour after filtration allowing the micro-dosed radiotracer to be used in humans up to one hour after filtration. We performed a preclinical evaluation program that included molecular diagnosis in myocardial ischemia-reperfusion rat model, acute oral toxicity in rats, mutagenicity in bacteria, biodegradability, extended single dose toxicity study in rats, and CARPA test in a hypersensitivity model in pigs. The objectives of this preclinical program were to establish the efficacy and safety profile that, if positive, would warrant fucoidan’s clinical evaluation in healthy volunteers. Two hours after intravenous injection of ^99m^Tc-fucoidan in rats and four hours after their reperfusion, a clear focal uptake had been detected in all animals supporting that this micro-dosed radiotracer is a relevant highly sensitive SPECT imaging agent for in vivo detection of cardiovascular pathologies associated with P-selection expression. The toxicology regulatory results showed that the GMP batch: (i) had no observable toxic effect in rats following oral administration of a single dose at a level of 2000 mg/kg; (ii) was not mutagenic to *Salmonella typhimurium* under the reported experimental conditions; (iii) had a low biodegradation; and (iv) had no observed-adverse-side effects in rats up to 400 μg/kg (injected dose: 40 μg/volunteer). Finally, CARPA experiments proved that, in a wide range of doses, fucoidan had no immune reactive properties in pigs.

In conclusion, we have presented the formulation of a radiolabeled macromolecular assembly at nanoscale of a natural polysaccharide (fucoidan) and technetium-99m with a good safety profile that exerts a molecular diagnosis by scintigraphy of cardiovascular diseases with P-selection expression. The micro-dosed radiotracer is produced according to GMP standards and can be readily used in human subjects for a phase I clinical investigations (safety).

## Figures and Tables

**Table 1:** Composition of LMW fucoidan as active pharmacological ingredient (API).

Test	Acceptance Limits	API
Aspect	fine powder	Fine white powder
Color	white to pale yellow	
**Identification**		
Sulfates	positive	positive
Fucose	positive	positive
**Active compounds content**		
Sulfates	for information	21.7% m/m
Total fucose	for information	31.7% m/m
Total fucoidan	≥40% m/m on dried substance	53.4% m/m as is 58.0% m/m dried
**Composition**		
Total galactose	for information	2.8% m/m
Total xylose	for information	10.5% m/m
Total mannose	for information	3.7% m/m
Total uronic acid	for information	18.0% m/m
Average mw	5 to 10 kda	7.1 kda
Free sulfates	for information	0.1
Free fucose	≤5% m/m	<0.1% m/m
Loss on drying	≤15% m/m	8.0% m/m
Total ash	≤30% m/m	19% m/m
**Heavy metals**		
Cadmium	≤1.0 ppm	<0.2 ppm
Lead	≤5.0 ppm	<0.5 ppm
Mercury	≤0.1 ppm	<0.1 ppm
Arsenic	≤1.5 ppm	<0.5 ppm
Formaldehyde	≤200 ppm	170 ppm
Yield	for information	5% m/m
**Microbiological quality**		
tamc	≤10^2^ cfu/g	<10 cfu/g
tymc	≤10^2^ cfu/g	<10 cfu/g
**Specified micro-organisms**		
*Pseudomonas aeruginosa*	absence in 1 g	absence in 1 g
*Staphylococcus aureus*	absence in 1 g	absence in 1 g
*Bacillus subtilis*	absence in 1 g	absence in 1 g
*Escherichia coli*	absence in 1 g	absence in 1 g
Bacterial endotoxins	≤3 eu/g	<0.0125 eu/g

**Table 2:** Composition of GMP-grade LMW fucoidan (GMP batch).

Tests	Acceptance Criteria	Clinical Batch
**Characteristics**		
Appearance	white to off-white powder	white powder
Water content	≤5% m/m	2.3
Appearance of reconstituted solution in water	clear and colorless	clear and colorless
**Sub-visible particles in water**		
Particles ≥ 10 µm	≤6000 particles/container	18
Particles ≥ 25 µm	≤600 particles/container	<1
Identification fucose	the retention time of fucose peak identical to the standard solution	Positive
Assay of active substance	36 to 44 µg per vial	40.3µg
Degradation products	≤5%	<0.1%
**Microbiological quality**		
Sterility	no microbial growth	no microbial growth <0.5
Bacterial endotoxins	≤120 EU/vial	EU/vial (<0.0125/µg)

**Table 3:** Analytical results of stability study of fucoidan lyophilized powder technical batch at 25 °C/60% RH.

Test Criteria	Acceptance Criteria	Stability Time Point (months)				
		T0	T1 month	T3 month	T6 month	T12 month
appearance	white to off white powder	white powder	white powder	white powder	white powder	white powder
water content	≤5% m/m	2.3	2.2	2.0	2.1	1.7
appearance of the reconstituted solution in water	clear, colorless	clear, colorless	clear, colorless	clear, colorless	clear, colorless	clear, colorless
sub-visible particles	≥10 µm: ≤6000 per container	18	-	-	-	-
	≥25 µm: ≤600 per container	<1	-	-	-	-
assay of active substance: fucoidan content based on fucose content in the drug product, as analytical marker	36 to 44 µg/vial	40.28	43.97	41.30	38.95	39.76
degradation products: free fucose content	≤5% m/m	0.09	0.09	0.10	0.08	0.07
sterility	no evidence of microbial growth	no evidence of microbial growth	-	-	no evidence of microbial growth	no evidence of microbial growth
endotoxins	≤120 EU/vial	<0.5	-	-	-	-

**Table 4:** Analytical results of stability study of fucoidan lyophilized powder technical batch at 40 °C/75% RH.

Test Criteria	Acceptance Criteria	Stability Time Point (months)			
		T0	T1 month	T3 month	T6 month
appearance	white to off white powder	white powder	white powder	white powder	white powder
water content	≤5% m/m	2.3	2.2	2.0	2.1
appearance of the reconstituted solution in water	clear, colorless	clear, colorless	clear, colorless	clear, colorless	clear, colorless
sub-visible particles	≥10 µm: ≤6000 per container	18	-	-	-
	≥25 µm: ≤600 per container	<1	-	-	-
assay of active substance: fucoidan content based on fucose content in the drug product, as analytical marker	36 to 44 µg/vial	40.28	41.01	41.98	39.21
degradation products: free fucose content	≤5% m/m	0.09	0.09	0.09	0.07
sterility	no evidence of microbial growth	no evidence of microbial growth	-	-	no evidence of microbial growth
endotoxins	≤120 EU/vial	<0.5	-	-	-

**Table 5:** Radioactivity and radiochemical purity of GMP-grade LMW fucoidan.

Tests	Acceptance Criteria	GMP-Batch_A	GMP-Batch_B	GMP-Batch_C
pH	6.5–7.5	7	7	7
radioactivity	400–800 MBq	758	546	592
**Radiochemical purity**				
MEK	(A) <5%	2	3	3
ACD-A	(B) <5%	2	2	1
RCP	>90%	96	95	96

## Supplementary Materials

The following are available online at https://www.mdpi.com/1660-3397/17/12/699/s1, Figure S1: Time course of cardiovascular (PAP, SAP and HR) changes following fucoidan and zymosan i.v. injections in pigs. Figure S2: Refractive Index (blue line) and Light Scattering (red line) chromatograms of purified fucoidan eluted in 0.1 M LiNO3 at 0.5 mL/min with Shodex SB-802.5 and SB-803 columns [22]. Figure S3: Scheme of extemporaneous preparation of radiolabeled GMP-grade LMW fucoidan (Investigational Medicinal Product). Figure S4: ITLC-SG plates spotted with fucoidan-FITC. UV exposition: Control (Left), MEK (Center), ACD-A (Right) Table S1: Composition of radiolabeled GMP-grade LMW fucoidan (Investigational Medicinal Product).
