# Supplementary material for: Pharmaceutical Development and Safety Evaluation of a GMP-Grade Fucoidan for Molecular Diagnosis of Cardiovascular Diseases
Source: Mar Drugs. 2019 Dec 12;17(12):699. doi: 10.3390/md17120699 (PMC6949921; doi:10.3390/md17120699)
Supplement: Supplementary file 1 [file marinedrugs-17-00699-s001.pdf]

Supplementary Materials

Pharmaceutical development and safety evaluation of a GMP-grade Fucoidan for molecular diagnosis of cardiovascular diseases

Cédric Chauvierre[1,*], Rachida Aid-Launais[1,2], Joël Aerts[2,3], Frédéric Chaubet[1], Murielle Maire[1], Lucas Chollet[1,4], Lydia Rolland[4], Roberta Bonafé[5], Silvia Rossi[5], Simona Bussi[5], Claudia Cabella[5], Laszlo Dézsi[6], Tamas Fülöp[6], Janos Szebeni[6], Youssef Chahid[7], Kang H. Zheng[7], Erik S.G. Stroes[7], Dominique Le Guludec[1,2,3], François Rouzet[1,2,3] and Didier Letourneur[1]

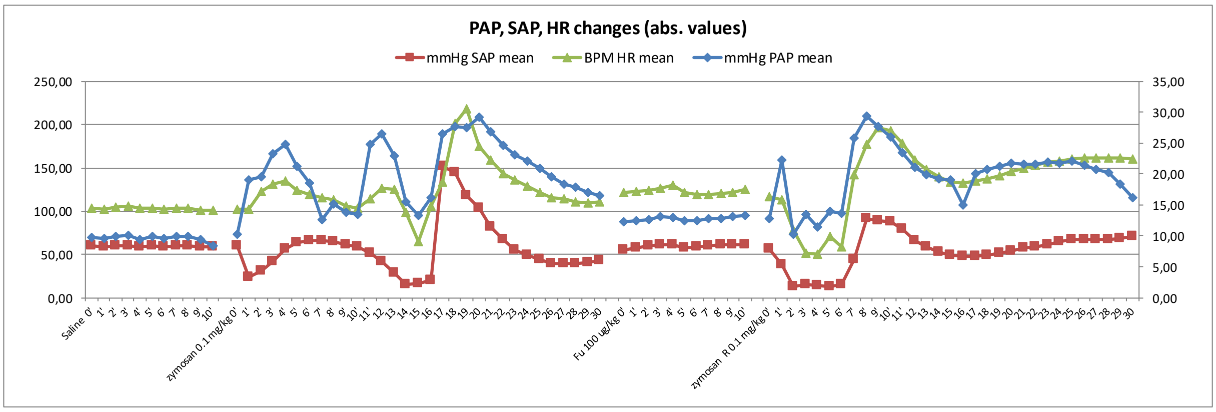

Figure S1: Time course of cardiovascular (PAP, SAP and HR) changes following fucoidan and zymosan i.v. injections in pigs.

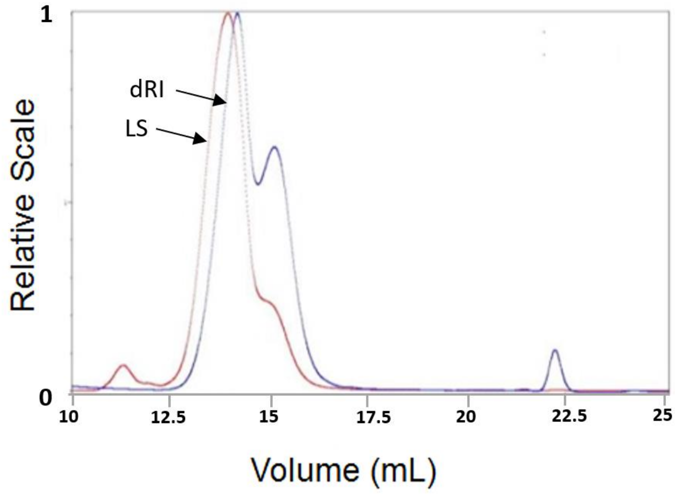

Figure S2: Refractive Index (blue line) and Light Scattering (red line) chromatograms of purified fucoidan eluted in 0.1 M LiNO3 at 0.5 mL/min with Shodex SB-802.5 and SB-803 columns [22].

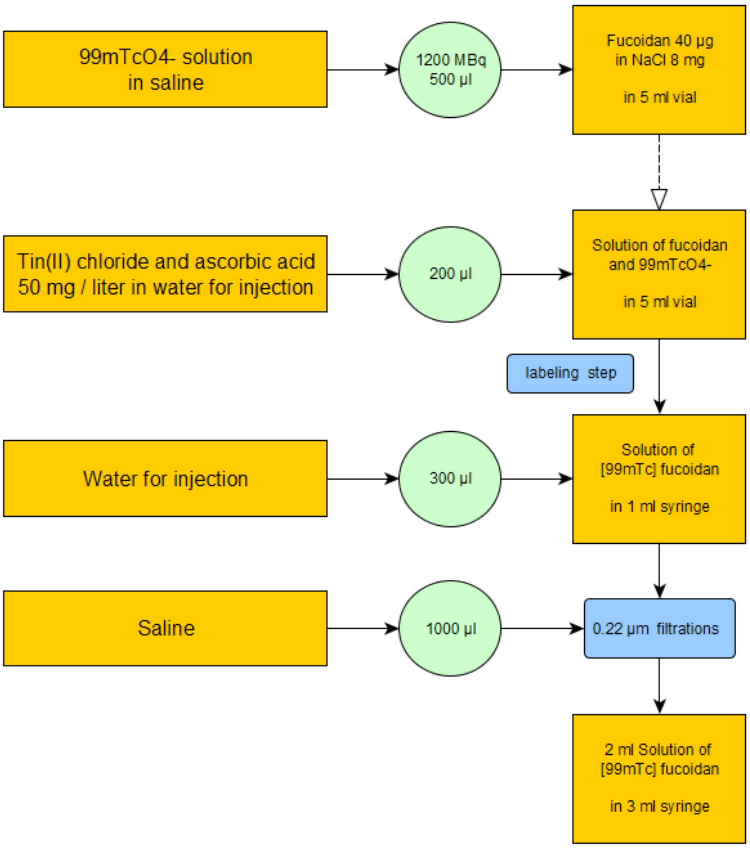

Figure S3: Scheme of extemporaneous preparation of radiolabeled GMP-grade LMW fucoidan (Investigational Medicinal Product).

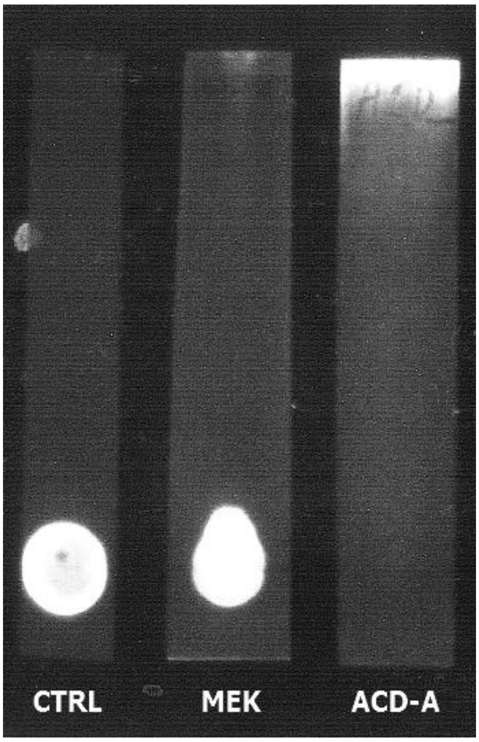

Figure S4: ITLC-SG plates spotted with fucoidan-FITC. UV exposition: Control (Left), MEK (Center), ACD-A (Right)

Component	Function	Quantity	Reference to Standards
Fucoidan, lyophilised powder	API	40 µg	In-House
^{99m}Tc sodium pertechnetate as Drytec® (Mo99/Tc-99m generator)	Radioactive precursor	1000-1300 MBq*	Authorized product<br>MA n° 564.454-1<br>(GE healthcare)
Stannous chloride dihydrate**	Reducing agent	12 µg	Ph. Eur.
Ascorbic acid	Antioxidant	10 µg	Ph. Eur.
NaCl***	Isotonicity agent	21.5 mg	Authorized product<br>Isotonic solution<br>for dilution NaCl 0.9%<br>MA n°351.159-6 (Braun)
Water for injection	Solvent	2 mL	Authorized products<br>340093-706.433-0 (Aguettant)<br>340093-585.515-2 (Baxter)

* Resulting in 400-800 MBq in the final labelled product

** Corresponding to 10 µg of anhydrous stannous chloride

***Coming from fucoidan lyophilized powder (8 mg), saline solution of Tc-99m (4.5 mg) and saline solution used during the preparation process

Table S1: Composition of radiolabeled GMP-grade LMW fucoidan (Investigational Medicinal Product).
